# Transforming food systems: a gendered perspective on local agricultural innovation in Cuba

**DOI:** 10.3389/fsoc.2023.1256379

**Published:** 2023-10-06

**Authors:** Bárbara Benítez Fernández, Erin Nelson, Anaisa Crespo Morales, Rodobaldo Ortiz Pérez, Rosa Acosta Roca, Regla María Cárdenas Travieso

**Affiliations:** ^1^Department of Sustainable Agroecosystem Management, National Institute of Agricultural Sciences (INCA), San José de las Lajas, Cuba; ^2^Department of Sociology and Anthropology, University of Guelph, Guelph, ON, Canada; ^3^Psychology Department, University of Pinar del Río (UPR), Hermanos Saiz Montes de Oca, Pinar del Rio, Cuba; ^4^Department of Genetics and Plant Breeding, National Institute of Agricultural Sciences (INCA), San Jose de las Lajas, Cuba

**Keywords:** participatory plant-breeding, gender, Cuba, local agricultural innovation, community development

## Abstract

Compared to many countries, Cuba has made significant progress in advancing women's rights and gender equity; however, disparities remain. In the country's rural communities and agricultural sector, women continue to face barriers to equal participation and recognition for the value of their work. This case study shares the story of gender equity efforts that have been conducted within the framework of a broader development project—the Project to Strengthen a System of Innovation in Local Agricultural Development (PIAL, for its initials in Spanish). PIAL began in 2001 as a participatory plant-breeding initiative aimed at increasing the genetic diversity of key crops such as maize and beans. Over the course of two decades, the project's goals expanded to include an emphasis on increasing women's participation. In the beginning, those efforts focused on including women in the participatory plant-breeding activities, which enabled them to prioritize traits they cared about such as grain texture, cooking speed, and taste in the selection process. Over time, the participatory nature of the PIAL methodology empowered women to identify and pursue capacity-building in other areas of local agricultural innovation. While PPB remained central to PIAL, women also chose to pursue opportunities in seed bank management, leadership training, and small-scale farm-based entrepreneurship. The results of the PIAL work on gender have included not just more inclusive plant breeding, but also important economic improvements for rural women as they have been able to diversify their livelihoods, and social change as they have gained confidence and recognition as leaders in their households, communities, and beyond.

## 1. Introduction

Compared to many nations in the Global South, Cuba is characterized by relatively high levels of women's participation across a range of activities. Policies and attitudes have helped advance gender equity in the economic sphere and with respect to domestic and care responsibilities. Still, inequities remain, as patriarchal cultural norms and structures are deeply rooted and continue to exert problematic influences, particularly for women (Bock and Shorthall, 2017). In the agricultural sector, significant gaps in gender equity continue to exist, and must be addressed in favor of women, who have been historically disadvantaged. For example, many women would benefit from training and capacity-building in productive activities, including crop and variety characteristics, management practices, and post-harvest conservation (Munster, 2017).

Given existing gender disparities in the country's agricultural sector (Cárdenas et al., 2017), it was necessary to establish gender policies that focused on equitable relations between women and men. The National Institute of Agricultural Sciences (INCA, for its initials in Spanish) and the Project to Strengthen a System of Innovation in Local Agricultural Development (PIAL, for its initials in Spanish) implemented a gender mainstreaming strategy with the aim of offering new opportunities for women's development that could serve as a tool to implement and evaluate a gendered approach in Cuban agricultural contexts (United Nations Development Program, 2019). The work to address gender within PIAL began with efforts to include women in participatory plant-breeding; however, over time, women identified their own priorities, and the scope of activities expanded into many other areas of agricultural innovation and sustainable community development.

Throughout the process, there was an emphasis on both individual change and collective action, creating space for radical changes in social thinking along with a transformation in social structures (Benítez et al., 2012). This case study report shares the story of how gender was mainstreamed into the PIAL framework, what activities and methods were employed, what key impacts were achieved, and what lessons were learned.

## 2. PIAL and it's evolving gender strategy

### 2.1. Phase 1 (2001–2006)

**Figure 1:**
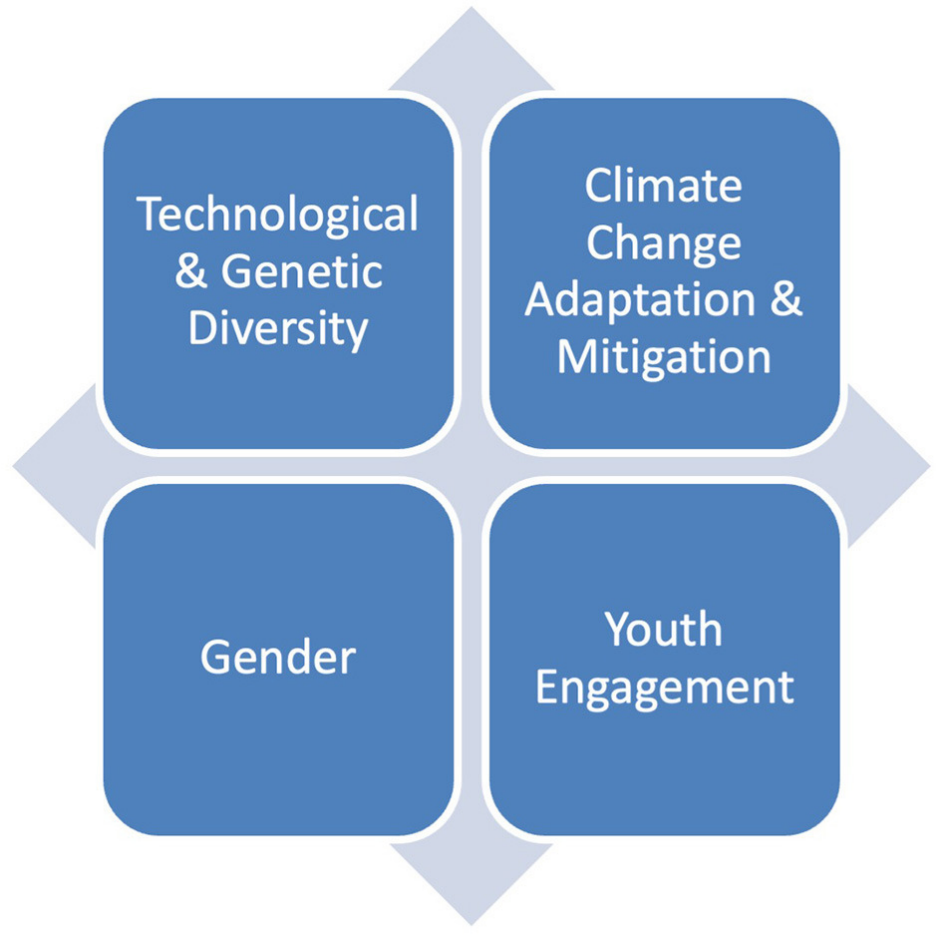
Four cross-cutting axes of PIAL in its later phases.

In 2001, INCA developed a Participatory Plant Breeding (PPB) project that aimed to take the important genetic resources located within the institute and place them in the hands of farmers throughout the country. With support from several research centers and national and international funders, that project continued for more than 20 years as researchers, farmers, and other stakeholders employed a Participatory Action methodology to increase the diversity and availability of various crops (mainly grains) on Cuban farms, household gardens, and other plots of land in 75 municipalities across 12 provinces (see Figure 1).

The primary mechanism driving this diversification has been *Ferias de Diversidad* (Diversity Fairs), wherein farmers grow multiple varieties on their farms and invite others to see the results and select their preferred seeds. These Fairs were important as many Cuban farmers were not connected to formal seed improvement programs run by research centers such as INCA and thus did not have ready access to improved varieties (Ortíz et al., 2015). Through the Diversity Fair system, varieties that had been developed by INCA plant breeders were shared with farmers to be grown out on their farms and further adapted and improved. To date, more than 680 such Fairs have been hosted by PIAL. The objective of this work is to ensure that a diversity of species and varieties of economic importance to the country are available to Cuban farmers. Project results quickly showed that the participatory approach led to an increase in both yields and genetic diversity on the farm and to significant social recognition for farmers when they selected their own varieties and shared them via the Fairs (Benítez et al., 2012).

The starting point for the varietal selections that took place via the Diversity Fairs was improved varieties that had been developed by plant breeders at INCA. These breeders manage research programs designed to improve varieties of interest to the Ministry of Agriculture and of importance to the country for economic and food security reasons. PIAL facilitated enhanced collaboration between these institution-based breeders and Cuban farmers. As the project advanced, improved varieties developed through INCA's breeding programs were introduced into the Diversity Fairs to gauge their acceptance by Cuban farmers and to enable those farmers to continue their farm-based breeding efforts, sharing results back to INCA. This reciprocal collaboration helped ensure that breeding efforts better reflect the diversity of climate and agricultural contexts across Cuba and farmer preferences and priorities. Notable plant breeding successes have included the development by INCA breeders of the Odile bean variety (among others), the Felo maize variety (among others), and multiple varieties of rice, soybean, and tomato (see Acosta et al., 2007; Ortiz et al., 2007; Cárdenas et al., 2021; Escalona et al., 2023). In two cases, farmers engaged in PPB through PIAL developed their own maize varieties, crossing varieties that had been shared with them through Diversity Fairs by INCA breeders.

Although women did participate in Diversity Fairs in the project's first years, their engagement in varietal selection was minimal; rather, they tended to play secondary roles, such as preparing meals and supporting event organization and logistics. Research by Verde et al. (2003) found this was largely due to ongoing gendered divisions of labor, wherein women's role in “productive” activities (including plant breeding) was subordinated to their role in “reproductive” activities (primarily related to the family and household), and their decision-making power relative to their male counterparts was limited. This is consistent with research demonstrating that, around the world, women's role in agricultural production is frequently under-recognized and under-valued (see Bock and Shorthall, 2017; Moyles, 2018; Bezner Kerr et al., 2019) and they are often excluded from agricultural research and development efforts (Bezner Kerr, 2017).

The study by Verde et al. (2003) underscored anecdotal awareness amongst project leaders that women's participation in the Diversity Fairs could and should be enhanced. As this awareness was growing, breeding efforts were also evolving. Whereas, initially the focus was almost exclusively on on rice (the production of which tended to be male dominated), the Diversity Fairs began to include beans, and then—in response to demand by participating producers—other grains such as corn, soybeans, and chickpeas, along with high-value crops such as vegetables and tubers, flowers, and ornamental plants. As this wider array of crops became integrated into the PIAL breeding activities, women's engagement in the Fairs became more active. The increasing involvement of women that occurred during this time was supported by project researchers and technicians—along with municipal, Ministry of Agriculture, and Communist Party of Cuba officials—who actively tried to recruit women into breeding and selection activities, receiving financial support from the Swiss Agency for Development and Cooperation (COSUDE) for those efforts.

A key component of women's integration into breeding activities was to ensure that varietal selection did not just take place in the fields but also through tasting dishes prepared using different varieties. This enabled attentiveness to traits that were of special importance to women, such as cooking quality and taste. As women became more involved in the breeding, these traits began to be considered, along with more traditional indicators such as yield and pest resistance, by plant breeders and technicians connected to the project. During this time, women also began to collect genetic material and refrigerate it in their homes to preserve and multiply it. These storage efforts gradually expanded in scale and evolved into more formalized Local Seed Banks, which will be discussed in more detail below as women have played a defining role in their operation. Through their inclusion in Diversity Fairs and breeding activities as well as the seed storage efforts, by the end of Phase 1 of the project, women's participation had increased, although gender considerations had not yet been centered in the work.

### 2.2. Phase 2 (2007–2011)

As the PIAL project entered its second phase, a series of Gender Pilot Projects were initiated in four provinces (Pinar del Río, Havana, Villa Clara, and Holguín). These pilot projects sought to increase women's involvement in and capacity for agricultural activities, particularly those related to crop diversification and varietal selection, but also across a range of other areas identified by the women themselves (e.g., poultry management, and food preservation). More broadly, they aspired to empower rural women and transform their realities, and those of their families, facilitating attitudinal and material shifts in women's household and community roles (Benítez et al., 2020).

Women from each province were chosen and trained to serve as *referentes de género* (gender representatives) at the municipal, provincial, and national scales. These women—farmers, researchers, and other specialists—took part in intensive gender training emphasizing sensitization to gender focal points, empowerment, leadership, and women as agents of change. The methodologies employed for that work included Gender-Focused Participatory Diagnostics (see below for more detail) and Gender Indicator Development. The Gender Pilot Projects proved successful at enhancing women's engagement with PIAL. Building upon that success, in 2011, gender mainstreaming was formally identified as an essential cross-cutting axis for the PIAL project.

### 2.3. Phases 3 (2012–2016) and 4 (2017–2022)

During the latter phases of PIAL, gender became increasingly central, as exemplified in its identification as one of four foundational axes for the project, along with technological and genetic diversity, climate change adaptation and mitigation, and youth engagement (Romero et al., 2018). The explicit recognition of the synergies across these axes ensured that activities would jointly take into consideration making progress toward gender equity while also increasing the diversity of seed varieties, for example, by ongoing attention to women's active participation in seed-breeding, saving, and sharing processes.

Specific actions that supported attentiveness to gender included: dedicated funding for gender-focused project activities; inviting the person responsible for coordinating gender work to join the PIAL National Coordinating Group; permanently incorporating gender-focused research and training into the project framework; and developing new programming based on priorities identified by women. Some elements of the PIAL methodology in its later phases that illustrate the connectivity between gender and technological and genetic diversity include (see Benítez et al., 2020 for further detail):

1. **Gender-focused participatory diagnostics**. These will be discussed in more detail below.
2. **Capacity-building exchange workshops**. Women, youth, and men from the 10 provinces where PIAL actively participated in these workshops, which employed a social learning approach to foster new knowledge and skills in areas participants themselves identified as priorities. This included more traditional activities related to agricultural innovation, diversity, and sustainability, along with gender-specific topics such as hegemonic masculinities, self-esteem, and gender-based violence.
3. **Women-led Local Agricultural Innovation Groups**. These farmer-led groups (GIALs, for the initials in Spanish) are dedicated to on-farm experimentation and trials to develop and refine agricultural innovations, including but not limited to PPB. While women, youth, and men can lead a GIAL, since the implementation of the PIAL gender strategy, groups have increasingly been led by women, thereby ensuring women's agricultural research questions and innovation ideas are addressed.
4. **Micro-grants**. These competitive grants were awarded to PIAL participants for initiatives they proposed related to developing and/or sharing agricultural innovations and good practices. More than half of these grants went to women participants.
5. **Provincial, national, and international exchange visits**. The spaces created at multiple scales for exchange amongst women, men, and youth enable participants to gain valuable leadership experience as knowledge-sharers and capacity-builders. They also served as the basis for achieving greater crop diversification as information about multiple crops and newly developed varieties could be shared more widely amongst producers, decision-makers, and research technicians. All this brought with it an increase in diversity on farms, which resulted in a better quality of life for families (for more detail, see Benítez et al., 2020).
6. **Strategic Planning with a Gender Approach**. The project offered courses in strategic planning, which served as a vehicle for women and men to build strategic plans for strengthening economic initiatives related to, for example, crop production, food preservation, animal husbandry, orchard management, small and large livestock rearing, feedstock production, and flower and ornamental plant production. Project personnel made a concerted effort to include women in these courses, and gender parity across all participants was very nearly achieved.
7. **Multi-actor management platforms**. As PIAL began to focus on institutionalizing its processes, these platforms were developed to foster connectivity between the project and strategic institutional allies such as the National Association of Small Farmers (ANAP), the Federation of Cuban Women (FMC), the Ministry of Agriculture (MINAG), the Communist Party of Cuba (PCC), and a variety of research Institutes, universities, local governments, and local development projects.

## 3. Analysis: evolution of PIAL gender and participatory plant breeding efforts

### 3.1. Implementing gender-focused participatory diagnostics

An essential component that highlights how PIAL synergistically developed its gender-focused and PPB work is the implementation of Gender-Focused Participatory Diagnostics (DPEG, for its initials in Spanish) (Aguilar et al., 1999). This methodology aimed to simultaneously improve gender equity and strengthen local agricultural systems in communities where PIAL was active.

The implementation of DPEG as a core part of PIAL was, in part, a direct response to anecdotal recognition on the part of project technicians and specialists that women's participation in PPB processes had been relatively low. This inequitable participation represented a limitation for the project and was contrary to its goals of facilitating participatory processes and knowledge dialogues grounded in horizontal relationships amongst so-called “experts” and producers in rural communities. In striving for broad-based, horizontal participation, knowledge-sharing, and dialogue, it was clear that more rural women needed to be engaging with PPB processes. Conducting systematic gender-based diagnostics in a participatory manner was an important step toward achieving that goal.

A suite of gender analysis tools was introduced into PIAL through collaboration with researchers and specialists from various institutions (universities, research centers, Ministry of Agriculture centers), who were supported by training from an international expert in the field. Specific personnel charged with carrying out the gender analyses were elected by provincial project coordination teams. The tools included activities designed to identify, explore, and assess a wide range of gendered issues in farming communities, including gender roles, socio-economic and cultural positioning and condition of women and men, people's interests, priorities, aspirations, and needs, gaps in gender equity, access and control of resources, participation in family and farming (and other productive) decision-making, and engagement with formal and informal community organizations.

These activities were carried out using a combination of methods, such as participatory workshops, farm visits, and semi-structured interviews. Participants included female and male farmers, along with cooperative leaders, representatives of organizations such as the Federation of Cuban Women (FMC) and National Association of Small Farmers (ANAP), agricultural officials, and other rural and agricultural stakeholders. Importantly, the work was supported by the FMC, along with ANAP and governments, at multiple scales. It was guided by a “Gender and Development” approach, with the aim of analyzing power relations and supporting equitable redistributions of resources and power, in recognition of the theoretical and material importance of women's inclusion in global processes of economic, political, and social change (Romero and Ortiz, 2017). Initial DPEG results demonstrated the relatively disadvantageous situation of women in Cuban agricultural contexts.

### 3.2. Transforming women's role in Diversity Fairs

As mentioned above, Diversity Fairs are a central component of PIAL and serve as the fundamental method for the participatory selection of varieties of rice, bean, chickpea, soybean, tomato, cover crops, and other species. The DPEG work helped systematize and clarify some of the contributing factors that constrained women's participation in these Fairs during the project's first phase and point the way toward how their engagement might be enhanced.

The explicit incorporation of a gender approach in the project, beginning in 2007, was linked to a notable transition with respect to women's engagement in the Diversity Fairs. Not only did more women begin to participate, but the quality of their participation changed from playing a more passive role (e.g., meal preparation and other hosting and/or organizational activities) to engaging more directly and acting as participants in the PPB activities associated with the Fairs. Women's role in selecting preferred varieties proved important, as they brought new perspectives to the process, for example, prioritizing traits such as cooking quality, texture, and grain size and color, which had previously been overlooked as men focused on characteristics such as yields, and susceptibility to pests and disease.

The evolution of women's role in the Diversity Fairs speaks to the effectiveness of the DPEG efforts and the increasing emphasis placed on gender within PIAL, as the diagnostics and follow-up activities built awareness and capacity that favored women's active participation in PPB. Enhanced women's participation, in turn, ensured that the new varieties developed through PIAL would embody traits prioritized by women as well as men. It also contributed to women's self-esteem and facilitated their empowerment in multiple arenas beyond just the PPB work.

The transformation of women's engagement in the PIAL Diversity Fairs from relatively passive agents who fundamentally performed traditional reproductive roles to active agents of agricultural development and innovation offers an important illustration of women's capacity to act as agents of change in their communities. The gradual process of achieving this transformation was supported by the implementation of new forms of learning-in-action and the creation of new inter-institutional and human capacities to manage innovative processes, which are all essential to stimulate selection, maintenance, and dissemination of plant diversity (UNESCO, 2014).

### 3.3. Creating and consolidating local seed banks and local seed certification

At the same time, as a diversity of grain seeds, developed via PPB, was being introduced and disseminated in 45 municipalities across 10 provinces of the country, work was also being done to create the first PIAL Local Seed Banks. Because gender had already become a project consideration when the seed banks were initiated, there was attentiveness from the beginning to achieving equitable participation of women and men, with many women taking on leadership positions and working on selecting seeds to be stored.

Building upon early successes with seed bank development, in 2013, PIAL began to use “learning-in-action” cycles, wherein project personnel would work with community members to identify “bottlenecks” constraining development and innovation and collectively develop and implement strategies to address them. Like all PIAL work at the time, there was attention to gender as part of the process. In the first cycle, there was a strong emphasis on Seed Safety Diagnostics. In three municipalities representing the eastern, central, and western regions of the country (Bahía Honda, Manicaragua, and Gibara), a survey was conducted to identify gendered actions related to women's role in managing grain seed diversity and other locally important species. Results pointed to low participation of women as heads of farms, leading to calls to emphasize the role of women in the development of agricultural production systems, including production, processing, and marketing.

In 2016, a second learning-in-action cycle began, this time emphasizing production and local seed certification in 10 municipalities. This helped to consolidate and strengthen the Local Seed Banks, and the work on seed banks, seed safety, and local seed certification was expanded into all 75 municipalities where PIAL was active. At that time, the first Local Seed Certification Committees were created. Unlike the early days of the Diversity Fairs, women played active roles—including taking on leadership positions—in these committees from their inception, often serving as custodians of the Local Seed Banks, and were highly engaged in related activities.

The seed-focused learning-in-action work received significant support from the NGO USC Canada (currently SeedChange). There was also the active participation of the National Association of Small Farmers (ANAP), the Ministry of Agriculture (MINAG), the Ministry of Higher Education (MES), the Cuban Association of Agricultural and Forestry Technicians (ACTAF), and the Ministry of Science and Environment (CITMA). Importantly, this seed work was done in conjunction with the DPEG methodology described above. As such, there was ongoing attentiveness throughout the learning-in-action cycles to identifying, exploring, and assessing gendered roles, relationships, and power dynamics, and to working consciously toward gender equity in the activities.

### 3.4. Influencing public policy

As it evolved, PIAL explicitly sought, and secured, significant support from local governments. Buy-in from these authorities was important as local governments are responsible for local development within their municipalities. By forming strong alliances with these state bodies, social transformation processes were facilitated, wherein women played a key role in defining policies and processes, generating changes in favor of more equitable participation, empowerment, social recognition, improvement of self-esteem, and the generation of new opportunities for women and men in agricultural contexts. Integration of PIAL into local government structures also enabled good practices developed across the various axes of the project, including gender and technological and genetic diversity, to be institutionalized. One example of such institutionalization is that knowledge and methodologies generated through PIAL were incorporated into a research center and university curricula.

In addition to working with local governments, PIAL exerted policy influence at a national scale. For example, personnel from the project's national coordinating group worked in close collaboration with the Ministry of Agriculture and Federation of Cuban Women to develop a national equity strategy in the spheres of innovation and governance. This proved useful when the country had to respond to the COVID-19 pandemic. Part of the pandemic response involved boosting food production, and the equity strategy ensured that women and men from rural and urban areas were included in planning those efforts. A cornerstone of that work was the active promotion of family farming in patios and small plots to produce vegetables, medicinal plants, and herbs. Here, again, women played an important leadership role in producing fresh and safe food that positively impacted communities in a crisis.

PIAL also played a role in supporting Cuba's broader (and well-known) policies related to the promotion of urban and peri-urban agriculture, particularly by bringing its gender emphasis to the Urban, Suburban, and Family Agriculture (AUSUF) Movement. PIAL personnel and other researchers and technicians from the country's National Institute of Agricultural Sciences, worked to increase and diversify vegetable production in urban and peri-urban patios and plots across three provinces. This work was done using the same participatory, gender-focused approach that had been introduced through PIAL in close coordination with local governments and Popular Councils. The use of that methodology saw women's participation in the urban and peri-urban agriculture efforts in those three provinces increase to 35%. The work also incorporated PIAL expertise regarding seed conservation and handling, the use of bioproducts, and other agroecological methods; thus, there were capacity-building efforts to ensure the food being produced would be safe.

## 4. Overview of key impacts

### 4.1. Economic and productive results

The joint work of researchers, specialists, and technicians, accompanied by the National Association of Small Farmers, the Federation of Cuban Women, decision-makers from local governments, peasant organizations, and others, allowed the introduction of diverse technologies in farming communities across the country. More than 100 unique income-generating initiatives were strengthened in the twelve provinces where PIAL was active, 35% of which were led by women participants in agricultural innovation processes. Some of these activities were directly related to PPB, as the new varieties that were developed created economic opportunities for women and men; however, people pursued other initiatives as well based on their own preferences and priorities, including sales of flower arrangements, preserves, dairy products, and artisanal crafts (which often incorporated seeds).

Although not all income-generating projects were *directly* tied to PPB, there was a strong link between people's ability to develop new economic opportunities and the increasing species and varietal diversity on their farms that was facilitated by the PPB process. Indeed, project evaluations demonstrated that, prior to engaging with PIAL, farming families typically had very low levels of species and varietal diversity in their agroecosystems. The diversification process enabled women and men to begin to see a wide range of new economic possibilities that they could pursue according to their individual interests. PIAL also supported these endeavors as the Diversity Fairs and later introduced Innovation Festivals and Local Seed Banks (in which women played a fundamental role), which became important sites for micro-industry development, as new entrepreneurs could exchange products and ideas and provide motivation and support to each other.

The income generated through PIAL activities significantly affected participants' economic well-being. For example, the research found that women engaging in PIAL-related income-generation projects earned an average of 500 Cuban pesos (CUP) over and above their regular monthly salary. In some cases, that amounted to almost doubling their income. Notably, at the same time, food costs were typically reduced as more diversified farming systems reduced dependency on food purchasing.

### 4.2. Sociocultural impacts: changing subjectivities

While the economic impacts for women engaged in PIAL activities were important, the sociocultural changes sparked by the project were equally if not more meaningful. These changes in attitudes, behaviors, and perceptions occurred for both women and men and could be identified at the individual, family, and community scales. Women saw significant increases in their self-esteem as a result of their active participation and leadership in PIAL, and they also gained greater socio-economic independence. This translated into meaningful changes in the roles they played in their family units and the quality of the relationships within and beyond those units. Men also experienced changes in their perceptions related to productive and reproductive roles, and their shifting perceptions supported enhanced gender equity. The project also worked with young people to help ensure that the next generation of rural Cubans are motivated to appreciate their agrarian culture and to imagine gender relations in new, more equitable ways.

The innovation spaces generated from the gender axis (Innovation Fairs and Festivals, Culinary Festivals, Flower Fairs) have also constituted a good practice, where women, men, youth, and children have been motivated, achieving effective social inclusion and broad-based citizen participation, where women systematically exhibit the different modalities in which they work to develop their manual, artisanal, floristic, and culinary capacities (Benítez et al., 2021).

## 5. Summary: lessons learned

Many lessons can be drawn from the PIAL experience integrating gender as a cross-cutting axis in its PPB and other activities. Some of these lessons are summarized briefly here:

1. Adopting a gender-focused approach that included a participatory diagnostic methodology (DPEG) based on participatory action research principles and practices made it possible to make visible and strengthen the incorporation of women from Cuban contexts in activities in the agricultural sector.
2. Beginning with a “Women in Development” approach and later transitioning to a “Gender and Development” approach enabled the inclusion of both women and men in project actions. This was essential for evaluating power and gender relations in families and communities.
3. Capacity-building accompanied by national and international specialists constituted a basic tool to achieve awareness, changes in attitude, and understanding that help implement the tools of the Innovation System, both for decision-makers, and female and male producers.
4. Incorporating this approach to the participatory selection of varieties constituted a key element for the inclusion of women in processes of local agricultural innovation. It enabled attentiveness to their preferred traits (e.g., cooking quality, grain size, texture, taste) in breeding efforts.
5. The creation of Local Seed Banks and Seed Certification Committees with the participation and leadership of women constitutes a strength for the local agricultural innovation system.
6. Having a strategy aimed at generating job opportunities has been a fundamental step for the social empowerment of women, enabling them to become leaders, make decisions, and earn income, which translates into an improvement in their quality of life and the well-being of their families.
7. Employing a gender-focused approach to characterizing local systems in economic, agricultural, and social terms made visible exclusions, inequities, and discrimination that had previously not been appreciated or fully understood in the agricultural sector.
8. Establishing micro-grants for the execution of gender-sensitive budgets was a strength in the project, facilitating capacity-building and leadership opportunities for women within agro-productive value chains.
9. Having the participation of women, men, youth, and children in the innovation spaces convened from the gender axis constituted opportunities for the exhibition of productions, the exchange of experiences, the commercialization of products, and the social recognition of producers.
10. Collaboration with organizations and institutions and the revitalization of processes with a gender perspective in the institutional sphere represents a strength for the development of local agricultural innovation.
11. Collaboration with the Ministry of Agriculture (MINAG), in synergy with the Federation of Cuban Women, constituted an opportunity to support the gender strategy in order to offer employment opportunities, income for women and men in the agricultural contexts of the country and also to boost food production.
12. The evaluation of the results through gender dimensions and indicators made it possible to visualize changes as well as continued disparities for women and within families.

## Data availability statement

The original contributions presented in the study are included in the article/supplementary material, further inquiries can be directed to the corresponding author.

## Ethics statement

Ethical approval was not required for the studies involving humans because in the Cuban context, there is no structure for formal ethical approval. The research was approved by the scientific advisory committees of the involved institutions. The studies were conducted in accordance with the local legislation and institutional requirements. Written informed consent for participation was not required from the participants or the participants' legal guardians/next of kin in accordance with the national legislation and institutional requirements because in the Cuban context, participants in research of this nature are not required to provide informed consent. Written informed consent was not obtained from the individual(s) for the publication of any potentially identifiable images or data included in this article because in the Cuban context, this is not standard practice.

## Author contributions

BB, AC, RO, RA, and RC contributed to conception, design, and execution of the work discussed in the case study. BB and EN wrote the first draft of the manuscript. All authors contributed to manuscript revision, read, and approved the submitted version.
